# Hospital utilisation and the costs associated with complications of ICD implantation in a contemporary primary prevention cohort

**DOI:** 10.1007/s12471-022-01733-4

**Published:** 2022-11-24

**Authors:** M. van Barreveld, T. E. Verstraelen, E. Buskens, P. F. H. M. van Dessel, L. V. A. Boersma, P. P. H. M. Delnoy, A. E. Tuinenburg, D. A. M. J. Theuns, P. H. van der Voort, G. P. Kimman, A. H. Zwinderman, A. A. M. Wilde, M. G. W. Dijkgraaf, DO-IT investigators, M. van Barreveld, T. E. Verstraelen, E. Buskens, P. F. H. M. van Dessel, L. V. A. Boersma, P. P. H. M. Delnoy, A. E. Tuinenburg, D. A. M. J. Theuns, P. H. van der Voort, G. P. Kimman, A. H. Zwinderman, A. A. M. Wilde, M. G. W. Dijkgraaf

**Affiliations:** 1 Department of Cardiology, Heart Centre, Amsterdam UMC, University of Amsterdam, Amsterdam, The Netherlands; 2 Department of Epidemiology and Data Science, Amsterdam UMC, University of Amsterdam, Amsterdam, The Netherlands; 3 Department of Epidemiology, University Medical Centre Groningen, Groningen, The Netherlands; 4 Department of Cardiology, Thorax centre Twente, Medisch Spectrum Twente, Enschede, The Netherlands; 5 Cardiology Department, St. Antonius Ziekenhuis, Nieuwegein, The Netherlands; 6 Department of Cardiology, Isala Klinieken, Zwolle, The Netherlands; 7 Department of Cardiology, Division of Heart and Lungs, University Medical Centre, Utrecht, The Netherlands; 8 Department of Cardiology, Erasmus MC, Rotterdam, The Netherlands; 9 Department of Cardiology, Catharina Ziekenhuis Eindhoven, Eindhoven, The Netherlands; 10 Department of Cardiology, Noordwest Ziekenhuisgroep, Alkmaar, The Netherlands

**Keywords:** Nationwide registry, Implantable cardioverter defibrillator, Complications, Health resources, Healthcare costs

## Abstract

**Introduction:**

Implantation of an implantable cardioverter defibrillator (ICD) is standard care for primary prevention of sudden cardiac death. However, ICD-related complications are increasing as the population of ICD recipients grows.

**Methods:**

ICD-related complications in a national DO-IT Registry cohort of 1442 primary prevention ICD patients were assessed in terms of additional use of hospital care resources and costs.

**Results:**

During a median follow-up of 28.7 months (IQR 25.2–33.7) one or more complications occurred in 13.5% of patients. A complication resulted in a surgical intervention in 53% of cases and required on average 3.65 additional hospital days. The additional hospital costs were €6,876 per complication or €8,110 per patient, to which clinical re-interventions and additional hospital days contributed most. Per category of complications, infections required most hospital utilisation and were most expensive at an average of €22,892. The mean costs were €5,800 for lead-related complications, €2,291 for pocket-related complications and €5,619 for complications due to other causes. We estimate that the total yearly incidence-based costs in the Netherlands for hospital management of ICD-related complications following ICD implantation for primary prevention are €2.7 million.

**Conclusion:**

Complications following ICD implantation are related to a substantial additional need for hospital resources. When performing cost-effectiveness analyses of ICD implantation, including the costs associated with complications, one should be aware that real-world complication rates may deviate from trial data. Considering the economic implications, strategies to reduce the incidence of complications are encouraged.

**Supplementary Information:**

The online version of this article (10.1007/s12471-022-01733-4) contains supplementary material, which is available to authorized users.

## What’s new?

- In a large multicentre cohort of real-world primary prevention implantable cardioverter defibrillator (ICD) patients, we showed that device-related complications occur in a significant proportion of patients and are associated with substantial clinical consequences and hospital costs.
- Mean hospital costs for different types of complications, per complication and per patient with one or more complications are reported as well as a national estimate of yearly costs associated with complications of ICD implantation for primary prevention.
- The cost data can be used for health economic modelling (research practice) of new cardiac implantable electronic devices for heart rhythm disorders and facilitate ranking of targets for prevention of ICD-related complications.

## Introduction

Implantable cardioverter defibrillator (ICD) therapy has been proven effective for primary prevention of sudden cardiac death due to ventricular tachycardia or fibrillation [1, 2]. In an ageing population this therapy will continue to be a major treatment modality. Despite their effectiveness, ICD implantations are associated with complications and the complication rate is substantial [3–6]. It has been demonstrated that ICD-related complications are more frequent in more complex devices than in single-lead devices [3, 7–9]. Also, rates observed in clinical practice are higher than in clinical trials. However, current economic assessments of ICDs do not take these higher rates into account, resulting in biased cost-effectiveness data [10, 11]. With varying cost-effectiveness ratios and different approaches to including costs resulting from complications in cost-effectiveness studies, an appropriate estimation of the costs of ICD-related complications is necessary [12].

The increase in ICD implantations and related increase in device-related complications have economic implications and may pose a challenge to the healthcare budget. Although several studies have described ICD-related complications and their associations with device type and patient clinical characteristics, little information is available on the impact of ICD-related complications on healthcare resource utilisation and the associated costs. Furthermore, these implications for healthcare should be acknowledged and it should be investigated whether the different ICD-related complications show differences in type, intensity or cost of care. Furthermore, the need for improved risk stratification of primary ICD indications has been emphasised and if better patient selection is possible, then these complication costs can be prevented too [3].

To better understand the economic impact of ICD-related complications and their associated management, this paper addresses the hospital provider costs associated with complications that occurred within 2 years after device implantation for primary prevention as observed in a national clinical practice registry.

## Methods

### The DO-IT Registry

Patients who received their first ICD for primary prevention of sudden cardiac death between September 2014 and June 2016 were prospectively enrolled in the DO-IT (Dutch Outcome in Implantable cardioverter defibrillator Therapy) Registry [13]. This ICD cohort was set up to identify patients who do not benefit from ICD therapy within 2 years after implantation by developing prediction models for ICD therapy and all-cause mortality. All 28 Dutch ICD-implanting hospitals participated and the registry was approved by all institutional review boards.

After obtaining the patients’ informed consent, baseline data were collected on demographics, medical history, diagnostics, left ventricular ejection fraction and implant-related data. Data regarding mortality, (in)appropriate ICD therapy and ICD-related complications were extracted from medical records during regular protocol-based follow-up. All registry data were extensively monitored. More details of the registry and baseline characteristics of the patients included have been published elsewhere [13], followed by the report on both prediction models [14].

### Outcome measures

The primary outcome measure for the current analysis was the hospital provider costs for any ICD-related complication. Complications were defined as any undesirable clinical occurrence related to the ICD implantation and function. These complications were further categorised as related to the lead, the pocket, an infection or other causes. Any patient having one of these complications at any time during the observation period was included in the analysis. The secondary outcome measure was the hospital provider costs per type of complication.

### Data collection

Device interrogation records, electrophysical procedure reports, the hospitals’ patient administration and patient medical records were used to determine the patients’ clinical course directly related to a complication. The following information was gathered: details of the complication, re-interventions, length of additional hospital stay, extra consultations, and extra diagnostic or laboratory procedures.

### Unit costs

Unit costs were obtained from the latest complete unit cost sheet from one of the participating major hospitals [15] and from the most recent Dutch manual on costing [16] in healthcare research. The latter was only applied to hospital care provider consultations and hospital admissions. Unit costs are shown in Table S1 (Electronic Supplementary Material). Unit costs from different years are expressed in euros for the reference year (2019) after price indexing with general consumer-price index figures for the Netherlands (Statistics Netherlands, access month July 2019).

### Statistical analyses

Baseline characteristics (Tab. 1) are presented as mean (standard deviation) or median (interquartile range, IQR) as appropriate for continuous variables. Categorical variables are reported as percentages. Mean costs were calculated as the sum of the products of the volumes of hospital care components as reported in the DO-IT Registry with their respective unit costs. Volumes of resources of the main hospital care components and their associated costs are reported in separate tables. In Table S2 (Electronic Supplementary Material) only the volumes of the surgical interventions are mentioned, because these are most costly and have more impact on the patient. However, in Tab. 2 the costs for clinical interventions are presented, including surgical and non-surgical ones. Comparisons of the costs per complication between various patient subgroups were performed using the non-parametric Mann-Whitney-Wilcoxon or Kruskal-Wallis test as appropriate for sex, initial implant device type and ischaemic cardiomyopathy. The economic impact of complications was determined by multiplying the average costs associated with complications, their 2‑year per patient incidence rates as reported in the DO-IT Registry and the yearly number of primary prevention ICD implantations performed in the Netherlands. Hence, the impact reflected incidence-based costs, attributing expenses for complications during the first 2 years of follow-up after device implantation to each implantation. No discounting for time preference was applied.

**Table 1:** Baseline characteristics of study cohort^a^

Baseline variables	Patients with a complication (*n* = 195)
Male gender (%)	131 (67)
Age (SD)	66.46 (10.62)
BMI (SD)	27.22 (4.86)
NYHA functional class I, II, III/IV (%)	25 (13), 127 (66), 41 (21)
Ischaemic (%)	110 (56)
LVEF (SD)	26.08 (5.96)
NS-VT (%)	27 (14)
Atrial fibrillation (%)	65 (34)
COPD (%)	25 (13)
Hypertension (%)	91 (47)
Diabetes mellitus (%)	52 (27)
Beta blocker (%)	169 (87)
Aldosterone antagonist (%)	89 (46)
Diuretic (%)	132 (68)
ACEi or ARB (%)	166 (85)
*Initial device implant*^*b*^	
Single chamber (%)	39 (20)
Dual chamber (%)	33 (17)
CRT‑D (%)	103 (53)
sICD (%)	20 (10)

*BMI* body mass index, *NYHA* New York Heart Association, *LVEF* left ventricular ejection fraction, *SD* standard deviation, *NS-VT* non-sustained ventricular tachycardia, *COPD* chronic obstructive pulmonary disease, *ACEi* angiotensin-converting enzyme inhibitor, *ARB* angiotensin II receptor blocker, *CRT‑D* cardiac resynchronisation therapy defibrillator, *sICD* subcutaneous implantable cardioverter defibrillator

^a^For comparisons of baseline characteristics between patients with a complication and patients without a complication, see Table S3 (Electronic Supplementary Material)

^b^Three patients had a single-chamber ICD as initial implant but during follow-up received a subcutaneous ICD; one patient initially received a dual-chamber ICD but during follow-up a subcutaneous ICD was implanted

**Table 2:** Mean costs (€) per complication type

Type of complication	Frequency (no. of patients)	Clinical re-interventions	Hospitali-sation days	Outpatient consultations	Diagnostics	Total
*Lead related*	*140 (122)*	* 3,962*	* 1,490*	*135*	* 213*	* 5,800*
Lead dislodgement	48 (47)	5,113	1,526	113	167	6,919
Lead dysfunction	19 (17)	5,428	1,630	238	198	7,494
No LV lead placement^a^	17 (17)	4,493	3,005	74	202	7,775
Pneumothorax	13 (13)	1,906	1,328	45	226	3,505
Perforation	7 (7)	7,130	2,683	162	953	10,929
Diaphragmatic stimulation	16 (16)	2,412	508	176	139	3,235
Twiddler’s syndrome	2 (2)	5,062	2,031	243	319	7,654
Inappropriate sensing	12 (12)	526	296	136	65	1,024
Venous thrombosis	6 (6)	0	254	173	217	644
*Infection*	* 25 (25)*	* 9,876*	*11,962*	*253*	* 801*	*22,892*
Pocket infection	13 (13)	9,881	4,137	353	198	14,569
Systemic infection	12 (12)	9,870	20,440	144	1,455	31,909
*Pocket related*	* 49 (49)*	* 1,030*	* 829*	*232*	* 100*	* 2,191*
Pocket pain	5 (5)	8,976	1,218	233	94	10,520
Haematoma or bleeding	29 (29)	192	1,033	267	75	1,566
Other pocket problem	15 (15)	0	305	165	152	622
*Other*	* 16 (14)*	* 3,804*	* 1,333*	*195*	* 288*	* 5,619*
Early battery depletion	1 (1)	13,862	1,015	97	54	15,029
Other^b^	15 (13)	3,133	1,354	201	304	4,992
*Total*	*230 (195)*	* 3,969*	* 2,476*	*173*	* 258*	* 6,876*

*LV* left ventricular, *CRT‑D* cardiac resynchronisation therapy defibrillator, *VT* ventricular tachycardia, *RV* right ventricular

^a^Placement of LV lead not possible in patients with CRT‑D indication

^b^Pericarditis (*n* = 5), malfunction during testing (*n* = 3), haemothorax (*n* = 1), adverse effects of antibiotics (*n* = 1), fever and increased infection parameters attributable to phlebitis (*n* = 1), shock impedance out of range (*n* = 1), sustained VT during implantation attributable to RV lead manipulation, requiring external cardioversion (*n* = 1), erroneous injection of chlorhexidine (*n* = 1), guidewire fracture leading to abandoning of distal part in venous branch (*n* = 1)

A two-sided *p*-value < 0.05 was considered statistically significant. Statistical analyses were performed with SPSS version 24.0 (IBM Corp., Armonk, NY, USA).

## Results

Inclusion and follow-up data were collected for 1,442 patients. During a median follow-up of 28.7 months (IQR 25.2–33.7; minimal follow-up 24 months) 230 complications occurred in 195 patients (13.5%). Baseline characteristics of this cohort are listed in Tab. 1. For a comparison between patients with or without complication(s), see Table S3 (Electronic Supplementary Material). No major differences between the patient groups were observed except for device type and use of angiotensin-converting enzyme inhibitors. Within the subgroup of patients with a complication, the median duration from implant to the first ICD-related complication was 172 days (IQR 15.50–503.25). Most frequent complications were lead related, followed by pocket-related complications, infections and other complications, with a respective patient incidence rate of 8.5%, 3.4%, 1.7% and 1%.

### Hospital utilisation and cost assessment

The mean and total number of surgical interventions, hospital admissions, outpatient consultations and laboratory or diagnostic procedures for each complication type and each complication category are shown in Table S2 (Electronic Supplementary Material). An ICD-related complication required surgical intervention in 53%, with an average additional use of hospital resources per complication of 0.61 surgical interventions, 3.65 hospitalisation days, 1.6 outpatient consultation visits and 5 diagnostic or laboratory procedures. Most surgical re-interventions occurred in patients experiencing a lead-related complication. However, on average, infections resulted in the most surgical interventions per patient. In terms of hospital resources used, (systemic) infections had the most impact, followed by complications related to the lead.

**Fig. 1:**
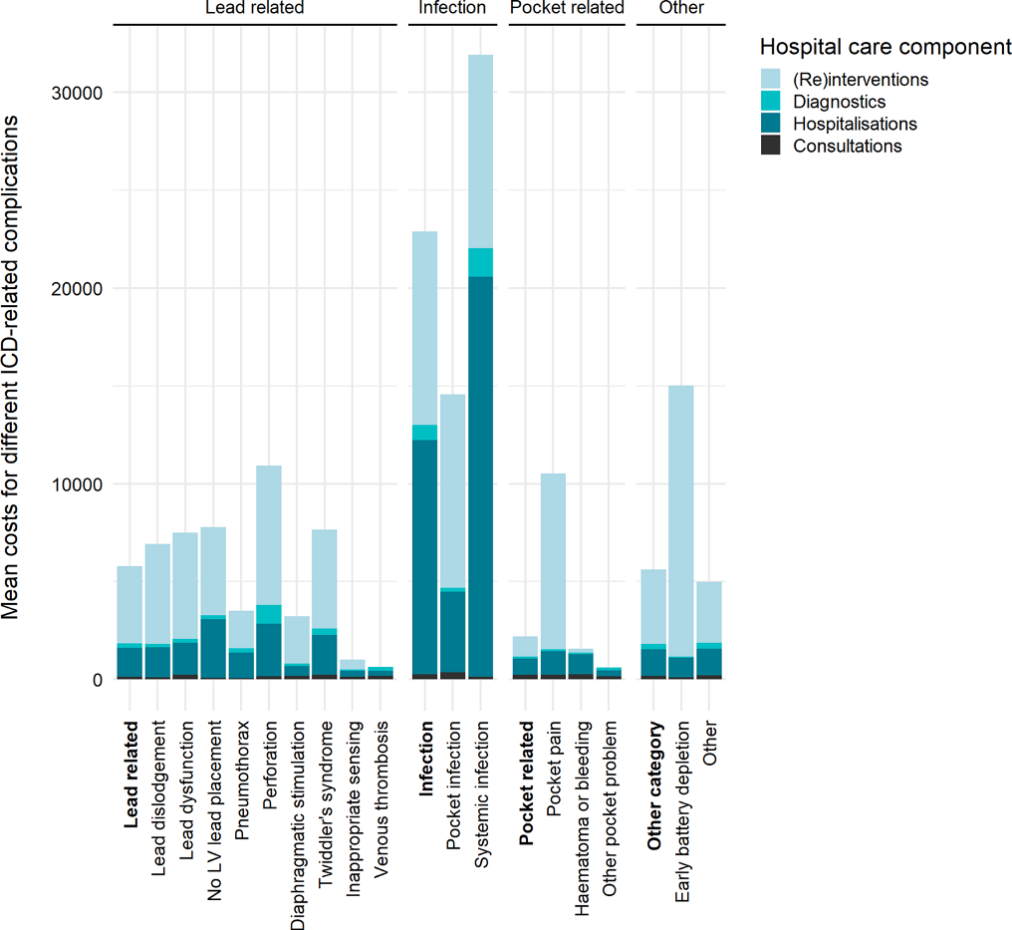
Mean costs per complication type per hospital care component. *ICD* implantable cardioverter-defibrillator, *LV* left ventricular

Tab. 2 shows the mean costs of the hospital care components stratified by complication type and category. Across all complication types the mean cost of management of an ICD-related complication was €6,876, to which the costs for clinical re-interventions contributed most (57%). Systemic infections were the most expensive complication, averaging €31,909 additional costs. These costs were primarily related to the extra hospitalisation days. Across all complications hospitalisation and clinical re-interventions were the main cost contributors (Fig. 1). Venous thrombosis and other pocket complications were least expensive. Per complication category, complications related to infection were most costly, but expenditures for hospital care utilisation for patients with a lead-related complication or complication due to other causes were also substantially higher than for patients with a pocket-related complication (€5,800 and €5,619 vs €2,190).

Complication costs were non-significantly higher in women than in men (€9,002 vs €7,675, *p* = 0.66). The mean costs per ICD-related complication in patients receiving a single-chamber ICD, dual-chamber ICD, CRT‑D and subcutaneous ICD were €6,825, €10,162, €8,267 and €6,425, respectively. Higher costs were observed in patients implanted with a dual-chamber or a CRT‑D device compared to those receiving a single-lead or subcutaneous ICD, but the difference was not significant. The costs resulting from an ICD-related complication for patients with ischaemic or non-ischaemic aetiology were similar (€7,815 vs. €8,338, *p* = 0.96).

### National hospital cost impact of complications

In the Netherlands, about 2,500 ICD implantations for primary prevention are performed every year [17]. An estimated 13.5% of these patients (338) are affected by an ICD-related complication during approximately the first 2 years after implantation. With the estimated mean costs of €8,110 per patient with one or more complications, this results in additional incidence-based costs of €2,741,180 per year. The cost impact per complication category is €1,407,683, €992,201, €186,119 and €155,870, respectively for complications related to the lead, infection, pocket or other causes. For more details on the cost impact per complication type, see Tab. 3.

**Table 3:** National cost impact (€) per complication type for 2,500 incident cases

Type of complication	Frequency	Observed patients	Clinical re-interventions	Hospitalisation days	Outpatient consultations	Diagnostics	Total
*Lead related*	*243*	*212*	* 961,645*	*361,630*	*32,742*	* 51,666*	*1,407,683*
Lead dislodgement	83	81	425,503	126,949	9,397	13,929	575,779
Lead dysfunction	33	29	178,803	53,690	7,826	6,537	246,857
No LV lead placement^a^	29	29	132,432	88,573	2,188	5,963	229,155
Pneumothorax	23	23	42,961	29,926	1,010	5,096	78,992
Perforation	12	12	86,534	32,566	1,967	11,562	132,629
Diaphragmatic stimulation	28	28	66,911	14,083	4,88	3,858	89,732
Twiddler’s syndrome	3	3	17,551	7,041	841	1,105	26,539
Inappropriate sensing	21	21	10,950	6,161	2,834	1,359	21,304
Venous thrombosis	10	10	0	2,640	1,799	2,256	6,695
*Infection*	* 43*	* 43*	* 428,057*	*518,473*	*10,955*	* 34,716*	* 992,201*
Pocket infection	23	23	222,707	17,131	7,952	4,455	328,353
Systemic infection	21	21	205,350	425,235	3,003	30,261	663,849
*Pocket related*	* 85*	* 85*	* 87,476*	* 70,413*	*19,722*	* 8,508*	* 186,119*
Pocket pain	9	9	77,805	10,562	2,019	811	91,197
Haematoma or bleeding	50	50	9,671	51,930	13,400	3,746	78,747
Other pocket problem	26	26	0	7,921	4,303	3,951	16,175
*Other*	* 28*	* 24*	* 105,517*	* 36,967*	* 5,396*	* 7,991*	* 155,870*
Early battery depletion	2	2	24,033	1,760	168	93	26,055
Other^b^	26	23	81,483	35,207	5,227	7,897	129,815
*Total*	*399*	*338*	*1,582,695*	*987,483*	*68,815*	*102,880*	*2,741,873*

*LV* left ventricular, *CRT‑D* cardiac resynchronisation therapy defibrillator, *VT* ventricular tachycardia, *RV* right ventricular

^a^Placement of LV lead not possible in patients with CRT‑D indication

^b^Pericarditis (*n* = 5), malfunction during testing (*n* = 3), haemothorax (*n* = 1), adverse effects of antibiotics (*n* = 1), fever and increased infection parameters attributable to phlebitis (*n* = 1), shock impedance out of range (*n* = 1), sustained VT during implantation attributable to RV lead manipulation, requiring external cardioversion (*n* = 1), erroneous injection of chlorhexidine (*n* = 1), guidewire fracture leading to abandoning of distal part in venous branch (*n* = 1)

## Discussion

This study focused on the additional hospital costs for management of complications after ICD implantation. Additional surgical re-intervention, hospitalisation days and use of diagnostic or laboratory measurements were common following a complication event. Quantification of hospital care utilisation and expenditures demonstrated that management of defibrillator complications is associated with significant costs. In our cohort 13.5% of patients experienced at least one ICD-related complication with an associated cost of €8,110 per patient or €6,876 per complication. As a consequence, in the costs for primary prevention defibrillator implantation not only the index hospitalisation for device implantation and subsequent monitoring costs should be taken into account, but additional mean costs of €1,095 per implant need to be considered to cover the costs related to complications within 2 years for the total cohort. Combining the complication costs and incidence rates per ICD type the mean additional costs are €555, €1,452, €1,367 and €1,179, respectively for a single-chamber, dual-chamber, CRT‑D or subcutaneous device implant. Infection of device systems was the most expensive complication, primarily resulting from additional hospitalisation days. However, not surprisingly other complications resulting in additional treatment days and surgical interventions also contributed to significant hospital costs.

This study provides insight into the economic burden of ICD-related complications. Costs related to complications should be incorporated into cost-effectiveness analyses and our results can be used as input for these economic evaluations. The cost-effectiveness analyses based on the findings of the primary prevention clinical trials were performed prior to the increase in implementation of more complex lead devices, which are associated with a higher complication rate in daily practice [3–6]. Therefore, the reported analyses may underestimate the real-world cost-effectiveness ratios. Furthermore, the possible decrease in patients’ quality of life due to a complication should also be taken into account.

Additionally, our findings report on the occurrence of ICD-related complications and their associated impact on patients in terms of clinical (surgical) interventions, outpatient consultations and additional hospitalisations. With one in seven patients experiencing an ICD-related complication, the complication rate in the DO-IT Registry is high compared to that in the landmark trials [1, 2]. However, our complication rate is in line with prior similar studies such as the DAI-PP (Defibrillateur Automatique Implantable-Prevention Primaire) and MADIT-CRT (Multicenter Automatic Defibrillator Implantation with Cardiac Resynchronization Therapy) [4, 18]. This higher complication rate is probably partly due to the comprehensive evaluation of complications in our study compared to large national registries that rely on administrative data with a risk of under-reporting. In addition, an underestimation of complication rates in randomised trials compared to actual clinical practice - because the trials were performed under ideal conditions with strict patient selection—may also contribute. Given this high complication rate and because ICDs are still the treatment of choice for this population, the search for strategies to reduce ICD-related complications is important. In larger studies risk factors could be detected for specific complications, as was done in the PADIT (Prevention of Arrhythmia Device Infection Trial) [19]. This might be useful for identifying circumstances that require more specific attention to optimise pre-implantation conditions to avoid ICD-related complications. However, as stated previously, more complex devices are currently implanted, which also contributes to the high complication rate. Moreover, the costs of these devices are also higher compared to single-lead devices. Therefore, from both a patient and economic perspective, treating physicians should carefully consider the choice of device type in each patient. Implanting a more complex defibrillator device by adding an atrial or left ventricular lead should only be done if a clear patient benefit is expected. Limiting unnecessary complexity of the device is an important first step in reducing ICD-related complications.

To estimate the national hospital cost impact, we performed an extrapolation of the economic implications due to ICD-related complications based on our own registry data. Because official data on the yearly number of primary prevention device implementations in particular are not available, a conservatively calculated yearly incidence of 2,500 patients was applied. Based on the reported average costs per complication type, extrapolation to other countries with purchasing power parities [20] is possible if the local data on incident cases or the incidences of ICD-related complications are available. With a yearly incidence of 2,500 primary prevention ICD implantations, the additional hospital costs for device-related complications are estimated at nearly 3 million euros per year. The overall societal costs, however, are likely to be even higher, because out-of-hospital healthcare costs, out-of-pocket expenses of patients and family members, costs resulting from productivity loss (40% of patients with an ICD-related complication aged ≤ 65 years) and intangible stress-related costs were not included.

This study has several limitations. First, these results may only be pertinent to the situation in the Netherlands, as we studied the hospital costs incurred as a result of all ICD-related complications in a large Dutch primary prevention cohort. With incidences and hospital care utilisation potentially being different in cohorts elsewhere, our results need further confirmation. In addition, the presented costs associated with ICD-related complications are likely an underestimation of the costs to society, since costs for out-of-hospital healthcare, out-of-pocket expenses and costs resulting from productivity loss could not be included. Second, our data were not sufficient to adequately report on the costs related to inappropriate shocks. However, with regard to the total costs associated with adverse consequences of defibrillator devices and the subsequent total costs of ICD implantation, the costs incurred due to inappropriate shocks are also relevant and should be addressed in future studies. Nevertheless, our findings indicate (data not shown) that the mean hospital costs per inappropriate shock were €884 (or €1,206 per patient), including the inappropriate shocks not resulting in hospital care. Third, the time horizon of the analysis was limited to approximately 2 years after device implantation; therefore, device replacements in the longer term and their subsequent complications and associated costs were not captured. Fourth, with data stemming from a registry there is a possibility of under-reporting in terms of complications and subsequent hospital care utilisation. In addition, 21 patients were lost to follow-up at some point in time and as a consequence complications may have been missed. No follow-up data were available for one patient, while seven patients emigrated during follow-up. A complication would be expected in approximately 3 patients (13.5% of 21 patients); however, during an average follow-up of 16.18 months we observed complications in 5 patients. Therefore, we think it is unlikely complications were missed and that this does not bias our results. Moreover, patient data were extensively monitored, and the relatively high complication rate suggests that under-reporting, if any, would have been minimal. The most dominant related in-hospital costs were accounted for. Rare use of related hospital resources (e.g. blood transfusion) was not included and co-medication for co-morbidities present was not recorded. Lastly, unit costs for clinical or surgical interventions and diagnostic and laboratory measures were partly based on the latest unit costing data of one of the participating major hospitals and may vary from one institution to another. Hence, the results should just be considered as strongly indicative.

In conclusion, data from this nationwide registry showed that additional hospital utilisation following ICD-related complications is substantial and treatment of complications may be expensive. This study demonstrates that complication-related costs can be an important component in the overall cost-effectiveness of device therapy. Our findings suggest that strategies to reduce the incidence of complications, reducing the costs of managing complications and sharpened ICD indications may result in significant reductions in hospital care utilisation and expenditures. Additionally, this financial assessment provides more accurate information on the cost implications of health outcomes and this information is of importance for reimbursement or hospital healthcare management.

## Supplementary Information

The Electronic Supplementary Material provides a list of all DO-IT investigators and supplemental tables to give readers additional information about their work
