# Supplementary material for: Hospital utilisation and the costs associated with complications of ICD implantation in a contemporary primary prevention cohort
Source: Neth Heart J. 2022 Nov 24;31(6):244–53. doi: 10.1007/s12471-022-01733-4 (PMC10188874; doi:10.1007/s12471-022-01733-4)
Supplement: Supplementary file 1 — The Electronic Supplementary Material provides a list of all DO-IT investigators and supplemental tables to give readers additional information about their work [file 12471_2022_1733_MOESM1_ESM.docx]

**Electronic Supplementary Material**

This supplementary material has been provided by the authors to give readers additional information about their work.

Supplement to: van Barreveld M, Verstraelen TE, Buskens E et al. Hospital utilisation and the costs associated with complications of ICD implantation in a contemporary primary prevention cohort

Last update: August 23, 2022

**Table of contents**

[List of DO-IT investigators .3](#_Toc37170976)

[Supplemental Tables 4](#_Toc37170977)

# **List of DO-IT investigators**

Listed in alphabetical order by institution.

Academic Medical Centre (A.A.M Wilde); Albert Schweitzer Hospital (M.W.F. van Gent); Amphia Hospital (S. Strikwerda); Canisius-Wilhelmina Hospital (L.H.R. Bouwels); Catharina Hospital (P.H. van der Voort); Erasmus Medical Centre (D.A.M.J. Theuns); Flevoziekenhuis (N.R. Bijsterveld); Isala Klinieken (P.P.H.M. Delnoy); Haga Hospital (R. Abels); Kennemer Gasthuis (R. Tukkie); Leiden University Medical Centre (L. van Erven); Maasstad Hospital (M. Firouzi); Maastricht University Medical Centre (K. Vernooy); Martini Hospital (L.H. Takens); Haaglanden Medical Centre (R.W. Grauss); Medical Centre Alkmaar (G.P. Kimman); Medical Centre Leeuwarden (A.E. Borger van der Burg); Medisch Spectrum Twente (M.F. Scholten); Onze Lieve Vrouwe Gasthuis (M. Khan); Rijnstate Hospital (R. Derksen); Scheper Hospital (M.W. Vet); St. Antonius Hospital (L.V.A. Boersma); Tweesteden Hospital (J.W.M.G. Widdershoven); University Medical Centre Groningen (A.H. Maass); Radboud Universiy Medical Centre (M.A. Brouwer); University Medical Centre Utrecht (M. Meine); Vlietland Hospital (H.A.M. Spierenburg); VU University Medical Centre (C.P. Allaart).

# **Supplemental Tables**

Table S1. Unit costs by type of resource

| **Health care components** | **Unit** | **Unit costs 2019 (euro)** | **Source** |
| --- | --- | --- | --- |
| **Inpatient care** |  |  |  |
| Inpatient days, cardiology | Day | 508 | DMC, 2014 |
| Inpatient days, intermediate care | Day | 1015 | DMC, 2014 |
| Inpatient days, intensive care | Day | 2149 | DMC, 2014 |
| **Outpatient consultations** |  |  |  |
| Cardiologist | Visit | 97 | DMC, 2014 |
| ICD technician | Visit | 97 | DMC, 2014 |
| Medical specialist | Visit | 97 | DMC, 2014 |
| General practitioner | Visit | 35 | DMC, 2014 |
| Emergency care | Visit | 276 | DMC, 2014 |
| Emergency heart care | Visit | 97 | DMC, 2014 |
| Home monitoring/ICD readout | Visit | 90 | UCS, 2017 |
| **Diagnostics** |  |  |  |
| Lab test NT-pro-BNP | Item | 23 | UCS, 2017 |
| Lab test haemoglobin | Item | 3 | UCS, 2017 |
| Lab test kidney function | Item | 8 | UCS, 2017 |
| Electrocardiogram | Item | 43 | UCS, 2017 |
| Chest X-ray | Item | 56 | UCS, 2017 |
| CT scan | Item | 146 | UCS, 2017 |
| Cardiac ultrasound | Item | 174 | UCS, 2017 |
| PET scan | Item | 323 | UCS, 2017 |
| CAG | Item | 1204 | UCS, 2017 |
| Phlebogram | Item | 116 | UCS, 2017 |
| Doppler ultrasonography | Item | 249 | UCS, 2017 |
| SPECT scan | Item | 266 | UCS, 2017 |
| MRI | Item | 428 | UCS, 2017 |
| Microbiological assessment | Item | 29 | UCS, 2017 |
| Stress electrocardiogram | Item | 167 | UCS, 2017 |
| **Clinical interventions** |  |  |  |
| Placement LV lead | Intervention | 5203 | UCS, 2017 |
| Placement other lead | Intervention | 5503 | UCS, 2017 |
| Lead replacement | Intervention | 5503 | UCS, 2017 |
| Lead reposition | Intervention | 4530 | UCS, 2017 |
| Lead extraction | Intervention | 4530 | UCS, 2017 |
| Single chamber ICD (re)placement | Intervention | 13862 | UCS, 2017 |
| Dual chamber ICD (re)placement | Intervention | 14847 | UCS, 2017 |
| CRT-D (re)placement | Intervention | 16561 | UCS, 2017 |
| SQ-ICD (re)placement | Intervention | 22229 | UCS, 2017 |
| ICD extraction | Intervention | 4530 | UCS, 2017 |
| ICD reposition | Intervention | 4530 | UCS, 2017 |
| Chest drainage | Intervention | 3540 | UCS, 2017 |
| Pericardial drainage | Intervention | 1626 | UCS, 2017 |
| Pocket drain | Intervention | 350 | UCS, 2017 |
| External cardioversion | Intervention | 422 | UCS, 2017 |
| Adjustment ICD settings | Intervention | 90 | UCS, 2017 |
| LifeVest | Month | 2000 | UCS, 2017 |

*ICD* implantable cardioverter defibrillator; *NT-pro-BNP* N-terminal pro B-type natriuretic peptide; *CT* computed tomography; PET positron emission tomography; *CAG* coronary angiogram; *SPECT* single-photon emission computerized tomography; *MRI* magnetic resonance imaging; *LV* left ventricular; *CRT-D* cardiac resynchronisation therapy defibrillator; *S-ICD* subcutaneous implantable cardioverter defibrillator.

Table S2. Mean and total volume of resources per complication type

| **Type of complication** | **Frequency**  **(*n* patients)** | **Surgical re-interventions %** | **Mean surgical interventions (total)** | **Mean hospitalisation days (total)** | **Mean outpatient consultations (total)** | **Mean diagnostics (total)** |
| --- | --- | --- | --- | --- | --- | --- |
| **Lead related** | **140 (122)** | **66** | **0.71 (99)** | **2.26 (317)** | **1.31 (184)** | **3.79 (530)** |
| Lead dislodgement | 48 (47) | 92 | 0.94 (45) | 2.46 (118) | 1.13 (54) | 3.63 (174) |
| Lead dysfunction | 19 (17) | 68 | 0.79 (15) | 2.58 (49) | 2.47 (47) | 3.84 (73) |
| No LV lead placement^*^ | 17 (17) | 82 | 0.88 (15) | 3.71 (63) | 0.76 (13) | 4.35 (74) |
| Pneumothorax | 13 (13) | 54 | 0.54 (7) | 2.46 (32) | 0.46 (6) | 4.08 (53) |
| Perforation | 7 (7) | 100 | 1.29 (9) | 3.57 (25) | 1.14 (8) | 9.86 (69) |
| Diaphragmatic stimulation | 16 (16) | 25 | 0.31 (5) | 1 (16) | 1.81 (29) | 3.25 (52) |
| Twiddler’s syndrome | 2 (2) | 100 | 1 (2) | 4 (8) | 2.5 (5) | 5 (10 |
| Inappropriate sensing | 12 (12) | 8 | 0.08 (1) | 0.25 (3) | 1.25 (15) | 1.15 (15) |
| Venous thrombosis | 6 (6) | 0 | 0 (0) | 0.5 (3) | 1.17 (7) | 1.67 (10) |
| **Infection** | **25 (25)** | **60** | **1 (25)** | **17.08 (427)** | **2.36 (59)** | **18.4 (460)** |
| Pocket infection | 13 (13) | 39 | 0.77 (10) | 6.7 (87) | 3.31 (43) | 5.15 (67) |
| Systemic infection | 12 (12) | 83 | 1.25 (15) | 28.33 (340) | 1.33 (16) | 32.75 (393) |
| **Pocket related** | **49 (49)** | **14** | **0.16 (8)** | **1.35 (66)** | **2.14 (105)** | **1.67 (82)** |
| Pocket pain | 5 (5) | 100 | 1.2 (6) | 2.4 (12) | 2.4 (12) | 2.8 (14) |
| Haematoma or bleeding | 29 (29) | 7 | 0.07 (2) | 1.62 (47) | 2.34 (68) | 1.66 (48) |
| Other pocket problem | 15 (15) | 0 | 0 (0) | 0.47 (7) | 1.67 (25) | 1.33 (20) |
| **Other** | **16 (14)** | **44** | **0.5 (8)** | **1.88 (3)** | **1.31 (21)** | **4.88 (78)** |
| Early battery depletion | 1 (1) | 100 | 1 (1) | 2 (2) | 1 (1) | 2 (2) |
| Other^†^ | 15 (13) | 40 | 0.47 (7) | 1.87 (28) | 1.33 (20) | 5.07 (76) |
| **Total** | **230 (195)** | **53** | **0.61 (140)** | **3.65 (840)** | **1.6 (369)** | **5 (1,150)** |

*LV* left ventricular; *CRT-D* cardiac resynchronisation therapy defibrillator; *VT* ventricular tachycardia; *RV* right ventricular

^*^Placement of LV lead not possible in patients with CRT-D indication

^†^Pericarditis (*n*=5), malfunction during testing (*n*=3),, haemothorax (*n*=1), adverse effects of antibiotics (*n*=1), fever and increased infection parameters attributable to phlebitis (*n*=1), shock impedance out of range (*n*=1), sustained VT during implantation attributable to RV lead manipulation, requiring external cardioversion (*n*=1), erroneous injection of chlorhexidine (*n*=1), guidewire fracture leading to abandoning of distal part in venous branch (*n*=1)

Table S3. Baseline characteristics of patients with and without an ICD-related complication

| **Baseline variables** | **Patients without a complication (*n*=1247)** | **Patients with a complication (*n*=195)** | **P-value** |
| --- | --- | --- | --- |
| Male gender (%) | 913 (73) | 131 (67) | 0.08 |
| Age (SD) | 65.75 (10.11) | 66.46 (10.62) | 0.37 |
| BMI (SD) | 27.34 (4.65) | 27.22 (4.86) | 0.74 |
| NYHA functional class I, II, III/IV (%) | 182 (15), 778 (63), 281 (23) | 25 (13), 127 (66), 41 (21) | 0.69 |
| Ischaemic (%) | 771 (62) | 110 (56) | 0.15 |
| LVEF (SD) | 26.06 (6.20) | 26.08 (5.96) | 0.97 |
| NS-VT (%) | 143 (12) | 27 (14) | 0.38 |
| Atrial fibrillation (%) | 373 (30) | 65 (34) | 0.27 |
| COPD (%) | 186 (15) | 25 (13) | 0.43 |
| Hypertension (%) | 526 (43) | 91 (47) | 0.32 |
| Diabetes mellitus (%) | 333 (27) | 52 (27) | 0.98 |
| Betablocker (%) | 1062 (85) | 169 (87) | 0.58 |
| Aldosterone antagonist (%) | 577 (46) | 89 (46) | 0.87 |
| Diuretic (%) | 899 (72) | 132 (68) | 0.20 |
| ACEi or ARB (%) | 1121 (90) | 166 (85) | 0.04 |
| Initial device implant^*^ |  |  | 0.00 |
| Single chamber (%) | 441 (35) | 39 (20) |  |
| Dual chamber (%) | 198 (16) | 33 (17) |  |
| CRT-D (%) | 519 (42) | 103 (53) |  |
| S-ICD (%) | 89 (7) | 20 (10) |  |

*BMI* body mass index; *NYHA* New York Heart Association; *LVEF* left ventricular ejection fraction, *SD* standard deviation; *NS-VT* non-sustained ventricular tachycardia, *COPD* chronic obstructive pulmonary disease; *ACEi* angiotensin-converting enzyme inhibitor; *ARB* angiotensin II receptor blocker; *CRT-D* cardiac resynchronisation therapy defibrillator; *S-ICD* subcutaneous implantable cardioverter defibrillator

^*^Three patients had a single-chamber ICD as initial implant but during follow-up received a subcutaneous ICD; one patient initially received a dual-chamber ICD but during follow-up a subcutaneous ICD was implanted
